# Intramolecular Metal Exchange Reaction Promoted by Thiol Ligands

**DOI:** 10.3390/nano8121070

**Published:** 2018-12-19

**Authors:** Yangfeng Li, Man Chen, Shuxin Wang, Manzhou Zhu

**Affiliations:** Department of Chemistry and Centre for Atomic Engineering of Advanced Materials, Anhui Province Key Laboratory of Chemistry for Inorganic/Organic Hybrid Functionalized Materials, Anhui University, Hefei 230601, Anhui, China; 18255127378@163.com (Y.L.); 13101@ahu.edu.cn (M.C.)

**Keywords:** alloy, metal exchange, atomically precise

## Abstract

The synthesis of an alloy nanocluster that is atomically precise is the key to understanding the metal synergy effect at the atomic level. Using the Ag_2_Au_25_(SR)_18_ nanocluster as a model, we reported a third approach for the metal exchange reaction, that is, intramolecular metal exchange. The surface adsorbed metal ions (i.e., Ag) can be exchanged with the kernel metal atoms (i.e., Au) that are promoted by thiol ligands. The exchanged gold atoms can be further stripped by the thiol ligands, and produce the Ag*_x_*Au_25−*x*_(SR)_18_^−^ nanocluster.

## 1. Introduction

Precise alloy nanoclusters have recently attracted great interest due to their quantum size effect-induced unique optical and catalysis properties [1,2]. More importantly, with their well determined structure, the relationship between their structure and properties can be grasped at the atomic level. Unlike for the large-sized gold nanoparticles (>3 nm), the method of synthesizing alloy nanoclusters is relatively limited. For instance, galvanic replacement is widely used in the synthesis of alloy nanoclusters. For example, the Ag-Pd alloy nanoparticle could be synthesized by reacting the silver nanoparticle with Pd(II) salt [3]. However, it is a challenge to synthesize an alloy nanocluster with this method, since the metal nanocluster, which is comprised by active metals, are quite unstable and rarely reported [4].

Previously, the metal exchange method, which uses homo-gold nanoclusters as templates to synthesize alloy nanoclusters, has been reported [5,6,7]. More importantly, the metal exchange process can jump out of the metal activity sequence, which provides a new way to synthesize the gold nanocluster with a highly active metal (e.g., Cd) [8,9]. So far, two types of metal exchange reactions have been reported: (i) Metal exchange with metal complexes [10,11,12,13,14,15]. This reaction can be created between thiolated metal complexes (e.g., Ag(SR)) and thiolated gold nanoclusters. (ii) Metal exchange between nanoclusters [16,17,18]. Recently, metal exchange was found between two nanoclusters. Pradeep and co-workers reported the reaction between Ag_25_(SR)_18_^−^ and Au_25_(SR)_18_^−^ to produce the Ag_x_Au_25−x_(SR)_18_^−^ nanocluster [16]. Based on these two methods, a series of alloy nanoclusters with atomically precise structures have been synthesized; meanwhile, such alloy products have been widely used to study metal synergistic effects in chirality, [19] optics [20,21], and catalysis [22] at the atomic level. It is still necessary to develop new alloying methods, especially a controllable alloying method.

In this work, we reported a third type of metal exchange: self-alloying induced by intramolecular metal exchange. Specifically, the intramolecular metal exchange reaction between Ag atoms and Au atoms in the Ag_2_Au_25_(SR)_18_ nanocluster can occur in the existence of thiol ligands and produce the Ag_x_Au_25−x_(SR)_18_^−^ nanocluster. The UV-Vis, thin layer chromatography, and mass spectra indicate the efficient transformation of this self-alloying process.

## 2. Materials and Methods

### 2.1. Materials

Tetrachloroauric(III)acid (HAuCl_4_·3H_2_O, >99.99% metals basis), silver nitrate (AgNO_3_, 99%, metal basis), sodium borohydride (NaBH_4_, 99.9%), phenylethyl mercaptan (PET, 99%), tetraoctylammonium bromide (TOABr, 98.0%), methylene chloride (CH_2_Cl_2_, HPLC grade), acetonitrile (MeCN, HPLC grade), methanol (MeOH, HPLC grade), and tetrahydrofuran (THF, HPLC grade) were purchased from Sigma-Aldrich (Shanghai, China). Pure water was purchased from Wahaha Co. Ltd., Hangzhou, Zhejiang, China.

### 2.2. Synthesis of the Au_25_(PET)_18_^−^ Nanocluster

The synthesis steps were similar to a previously reported process but with minor modifications [23]. HAuCl_4_·3H_2_O (0.2 g/mL, 0.4 mL) and TOABr (0.254 g, 0.47 mmol) was dissolved in 5 mL of THF. Then, 400 µL (2.8 mmol) of PET was added to the flask under a slow stirring speed ~60 rpm. After the solution turned clear (~30 min), the stirring speed was increased to fast (~1200 rpm). At the same time, an aqueous solution of NaBH_4_ (0.1550g, 4 mmol) was quickly added to the above solution to initiate the reaction. The reaction was allowed to proceed overnight. After that, the solution was washed with 5 mL pure water. The organic phase was dried via rotoevaporation. MeOH (~20 mL) was added to remove the byproducts and this centrifugation cycle was repeated at least 3 times. Then, 10 mL of MeCN was used to extract pure Au_25_(PET)_18_^−^TOA^+^ nanoclusters (NCs).

### 2.3. Synthesis of the Ag_2_Au_25_(PET)_18_^+^ Nanocluster

Ag_2_Au_25_ was prepared by modifying a previous method [24]. Briefly, 10 mg of Au_25_(PET)_18_^−^TOA^+^ (1.3 µmol) NCs was dissolved in 10 mL of MeCN. After that, 0.67 mg of AgNO_3_ (2.2 equivalents per mole of Au_25_), dissolved in MeCN, was added into the solution. The color of the solution then quickly turned from brown to dark green. The Ag_2_Au_25_ nanocluster was then precipitated out of the solution. MeCN (~10 mL) was added to remove the unreacted AgNO_3_ and other byproducts (e.g., TOA^+^NO_3_^−^).

### 2.4. Intramolecular Metal Exchange of the Ag_2_Au_25_ Nanocluster

2 mg of the Ag_2_Au_25_ nanocluster was dissolved in 10 mL of methylene chloride (DCM). After that, 0.1 mL PET was added into the solution. The color of the solution slowly turned from green to brown. This reaction was monitored by UV-Vis and MALDI-TOF-MS spectra. After ~70 min, all the Ag_2_Au_25_ nanoclusters were converted into Ag_x_Au_25−x_(SR)_18_^−^ (x = 0–2).

### 2.5. Characterization

All UV-Vis absorption spectra of the nanoclusters dissolved in CH_2_Cl_2_ were recorded using an Agilent 8453 diode array spectrometer (Shanghai China), whose background correction was made using a CH_2_Cl_2_ blank. The X-ray photoelectron spectroscopy (XPS) measurement was performed on a Thermo ESCALAB 250 (Waltham, MA, USA), configured with a monochromated AlKα (1486.8 eV) 150 W X-ray source, with a 0.5 mm circular spot size, a flood gun to counter charge the effects, and with the analysis chamber base pressure lower than 1 × 10^−9^ mbar. Inductively coupled plasma-atomic emission spectrometry (ICP-AES) measurements were performed on an Atomscan Advantage instrument made by Thermo Fisher (Waltham, MA, USA). The nanoclusters were digested by concentrated nitric acid and the concentration of the nanoclusters were set to 0.5 mg L^−1^ approximately. MALDI-TOF-MS was recorded on a Bruker Autoflex III smart beam instrument (Karlsruhe, Germany), using trans-2-[3-(4-tert-butylphenyl)-2-methyl-2-propenylidene] malononitrile (DCTB) as the matrix.

## 3. Results and Discussion

**Figure 1:**
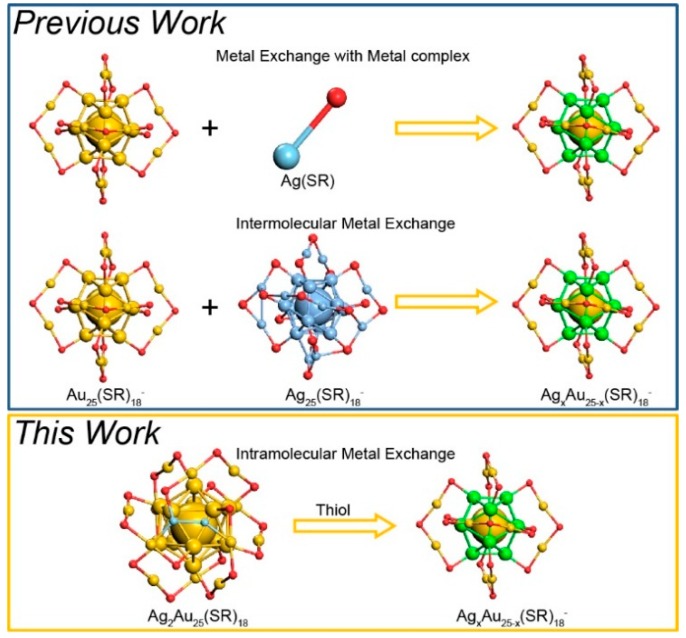
Synthesis of atomically precise alloy nanoclusters with different methods.

The Ag_2_Au_25_(SR)_18_ nanoparticle (Figure 1, abbreviated as Ag_2_Au_25_ hereafter) was made by reaction with the Au_25_(SR)_18_^−^ nanocluster, with two equivalents of AgNO_3_ in acetonitrile. We previously reported that the Ag(SR) complex reaction with Au_25_(SR)_18_^−^ (dissolved in toluene or dichloromethane) will produce the Ag_x_Au_25−x_(SR)_18_^−^ nanocluster [6]. However, by using the inorganic AgNO_3_ instead of Ag(SR), the silver atoms do not replace the gold atoms in the Au_25_(SR)_18_^−^ nanocluster but, instead, anchor on the surface of the Au_25_ nanocluster and produce the Ag_2_Au_25_ nanocluster. Interestingly, different kinds of metal salt precursors led to the different alloying result, that is, the metal-exchange or metal adsorption.

**Figure 2:**
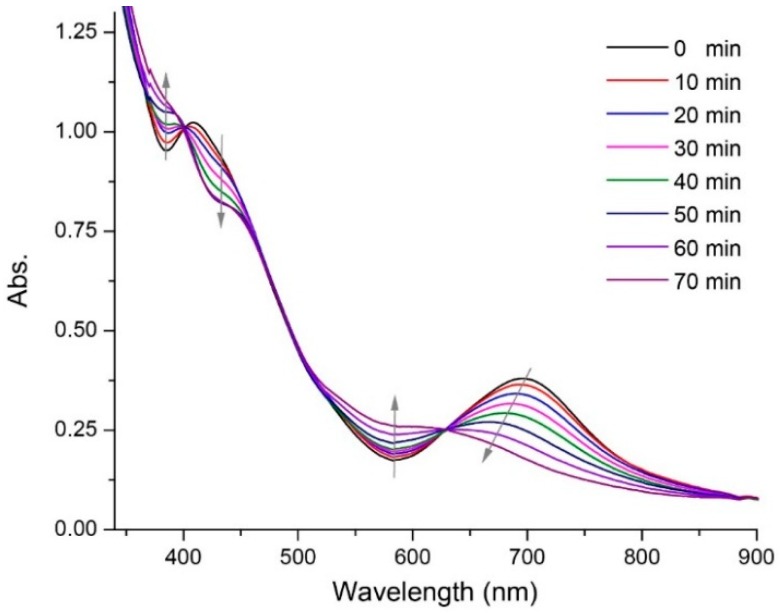
Time-dependent UV-Vis spectra of the Ag_2_Au_25_(SR)_18_ nanocluster reacting with PET ligands in dichloromethane.

**Figure 3:**
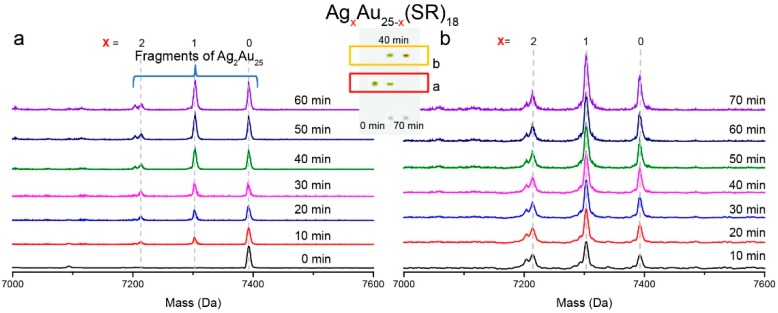
Time-dependent MALDI-TOF-MS spectra of the Ag_2_Au_25_(SR)_18_ reaction with PET. The inset picture is the Thin-layer chromatography (TLC) of samples at 0 min (Ag_2_Au_25_), 40 min, and 70 min, respectively. The positive model MALDI-TOF-MS results of the two components separated by TLC are shown in (**a**) and (**b**), respectively. Note that, the peaks in (**a**) are the fragments of Ag_2_Au_25_ according to [24]. The full spectrum of Ag_2_Au_25_ is shown in Figure S1.

We further reacted the Ag_2_Au_25_ with the thiol ligands. And applied the UV-Vis absorption spectra to monitor the reaction process. As shown in Figure 2, the HOMO-LUMO peak at ~700 nm of Ag_2_Au_25_ gradually blue shifted to ~625 nm. Meanwhile, three absorption points, at ~625 nm, ~475 nm, and ~410 nm, were found that indicated the quantitative conversion. The final product was determined to be Ag_x_Au_25−x_(SR)_18_^−^ with the *x* ranging from 0 to 2 (Figure 3b). The metal ratio was further confirmed by the XPS and ICP tests (Table 1).

Time-dependent TLC and MALDI-TOF-MS spectra were applied to monitor the reaction process (Figure 3). The TLC results showed the high purity of the Ag_2_Au_25_ nanocluster, after addition of the thiol ligands (PET) into the solution, the new compound was formed as demonstrated by the TLC. We further tested the mass spectrum of the two compounds, respectively. The mass spectrum suggested that the molecular ion peak of Ag_2_Au_25_ was not changed, whereas with the different fragment peaks, the pure Ag_2_Au_25_ nanocluster had one fragment peak at 7391 Da, which was caused by losing two Ag atoms at the surface. Interestingly, after the addition of the thiol ligands, we found three fragment peaks at 7391 Da, 7302 Da, and 7213 Da. The mass difference (89 Da) was equal to the mass difference between Au (197 Da) and Ag (108 Da). The different fragmentation after the addition of the thiol ligands suggested that the silver atoms at the surface were exchanged by gold atoms. The new compounds were determined to be Ag_x_Au_25−x_(SR)_18_ nanoclusters, which indicated the occurrence of the metal exchange process.

There are two main approaches in which this metal exchange can occur: (i) In direct metal exchange. In this way, thiol ligands first strip the adsorbed silver ions from the Ag_2_Au_25_ nanocluster. This process results in the Ag(SR) complex and the Au_25_(SR)_18_^−^ nanocluster. Then, metal exchange occurrs between these two products and results in the Ag_x_Au_25_(SR)_18_^−^ nanocluster. (ii) In intramolecular metal exchange, that is, adsorbed silver ions are exchanged with the kernel gold atoms, which are further stripped by thiol ligands. In order to study this reaction, the mass analysis was applied on the new compounds, which were separated from the TLC. The mass spectra showed similar results during the reaction. The absence of Ag_2_Au_25_(SR)_18_ indicated this composition had reacted. Three intense peaks, at 7391 Da, 7302 Da, and 7213 Da, were found, which were assigned to Ag_2_Au_23_, Ag_1_Au_24_, and Au_25_, respectively. It is worth nothing that, the ratio of the three peaks was maintained at 1:2:1 during the reaction. For comparison, we applied the metal exchange reaction between the Au_25_(SR)_18_^−^ nanocluster with the Ag(SR) complex; the time dependent mass spectra and UV-Vis absorption spectra are shown in Figure S2. Consequently, the intermolecular metal exchange reaction between Ag(SR) with Au_25_(SR)_18_^−^ was quite different from the present work. The proportion of doped silver atoms gradually increased with time. These results ruled out the idea that the metal exchange in the Ag_2_Au_25_ nanocluster resulted from the thiol ligand pulling out the adsorbed silver atoms, and the resultant intermolecular metal exchange reaction.

**Scheme 1:**
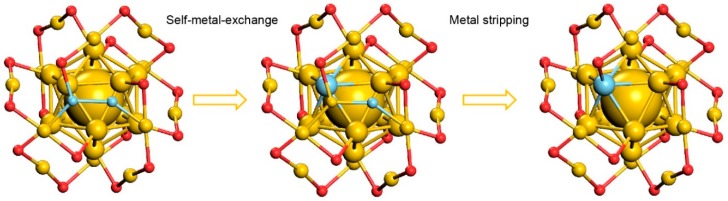
Th proposed intramolecular metal exchange process.

The intramolecular metal exchange in the Ag_2_Au_25_ nanocluster contained two steps (Scheme 1): (i)self-metal-exchange; (ii) metal stripping. In the first step, surface silver atoms exchanged the gold atoms at the kernel, with the promotion of the thiol ligands, and produced the isomeric Ag_2_Au_25_ nanocluster. In the second step, the surface atoms dissociated from the surface of the cluster and produced the Ag*_x_*Au_25-*x*_ nanocluster. The 1:2:1 ratio, which was found from the mass spectra of the final product, revealed that the silver to gold ratio was 1:1.

## 4. Conclusions

In summary, we reported the intramolecular metal exchange in the Ag_2_Au_25_ nanocluster. The intramolecular exchange involves two steps. First, silver atoms at the surface interchange with the gold atoms at the kernel, then the metal ions on the cluster surface are stripped from the cluster. With the intramolecular reaction, we obtained the Ag_x_Au_25−x_(SR)_18_^−^ (*x* = 0–2) nanocluster with a quantitative yield. Our work not only provides a new approach for the metal exchange reaction, but also provides a new perspective for understanding the metal behavior at the nanoscale.

## Figures and Tables

**Table 1:** The atomic ratio of Au:Ag in the Ag_x_Au_25−x_(SR)_18_^−^ nanocluster, calculated by Inductively coupled plasma-atomic emission spectrometry (ICP-AES) and X-ray photoelectron spectroscopy (XPS) measurements.

Results	Au Atom	Ag Atom
ICP experimental ratio	96.22%	3.78%
XPS experimental ratio	96.04%	3.96%
Theoretical	24/25 (96%)	1/25 (4%)

## Supplementary Materials

The following are available online at http://www.mdpi.com/2079-4991/8/12/1070/s1, Figure S1: The full spectra of Ag_2_Au_25_ nanocluster with different laser energy during the MALDI-TOF-MS analysis. The intensity of Au_25_ decreases with the decrease of laser intensity, which indicate the Au_25_ is the fragment of Ag_2_Au_25_; Figure S2: Time-dependent MALDI-TOF-MS spectra of metal exchange between Au_25_(SR)_18_^−^ and Ag(SR) (4 equivalents) complex. (a) 0 min; (b) 5 min; and (c) 15 min.
