# Supplementary material for: Intramolecular Metal Exchange Reaction Promoted by Thiol Ligands
Source: Nanomaterials (Basel). 2018 Dec 19;8(12):1070. doi: 10.3390/nano8121070 (PMC6316370; doi:10.3390/nano8121070)
Supplement: Supplementary file 1 [file nanomaterials-08-01070-s001.pdf]

Supporting Information

Intramolecular Metal Exchange Reaction Promoted by Thiol Ligands

Yangfeng Li, Man Chen, Shuxin Wang * and Manzhou Zhu *

Department of Chemistry and Centre for Atomic Engineering of Advanced Materials, Anhui Province Key Laboratory of Chemistry for Inorganic/Organic Hybrid Functionalized Materials, Anhui University, Hefei, Anhui 230601, China; 18255127378@163.com (Y.L.); 13101@ahu.edu.cn (M.C.)

* Correspondence: lxing@ahu.edu.cn (S.W.); zmz@ahu.edu.cn (M.Z.); Tel.: +86551-63861487 (S.W. & M.Z.)

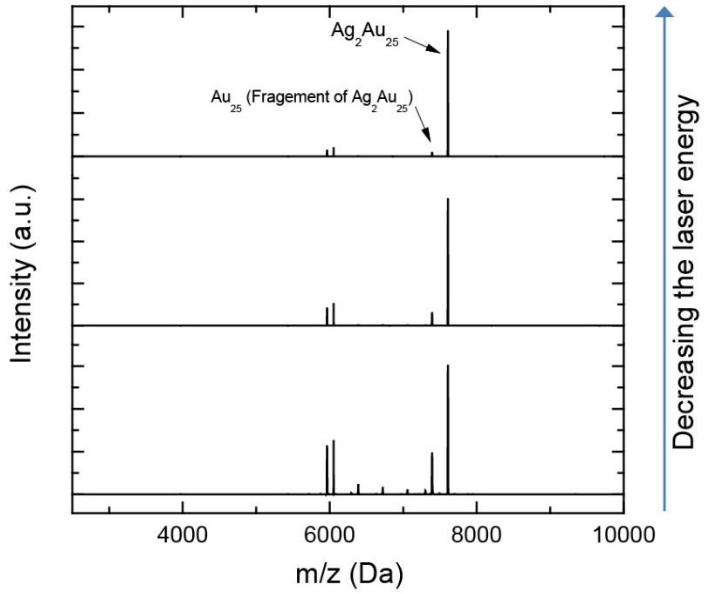

Figure S1. The full spectra of Ag_2Au_{25} nanocluster with different laser energy during the MALDI-TOF-MS analysis. The intensity of Au_{25} decreases with the decrease of laser intensity, which indicate the Au_{25} is the fragment of Ag_2Au_{25}.

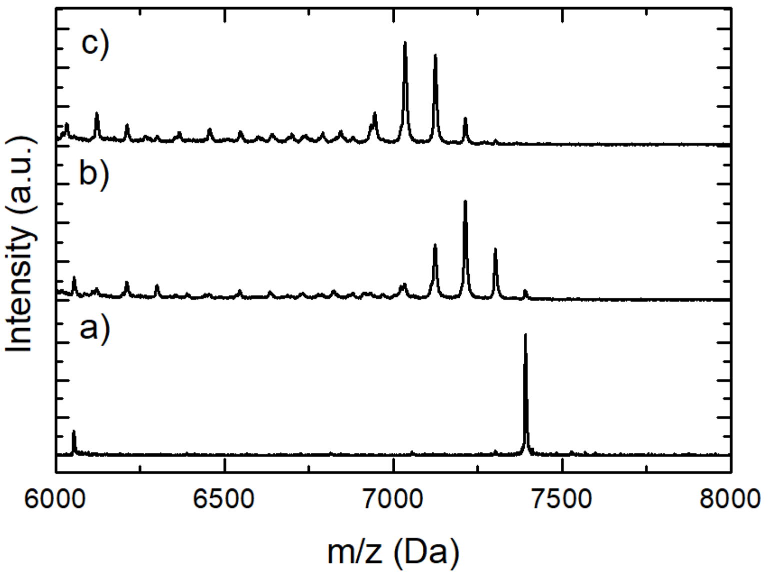

Figure S2. Time-dependent MALDI-TOF-MS spectra of metal exchange between $Au_{25}(SR)_{18}^-$ and Ag(SR) (4 equivalents) complex. (**a**) 0 min; (**b**) 5 min; and (**c**) 15 min.
